# [Retracted] MicroRNA‑136 inhibits prostate cancer cell proliferation and invasion by directly targeting mitogen‑activated protein kinase kinase 4

**DOI:** 10.3892/mmr.2023.13003

**Published:** 2023-05-02

**Authors:** Yudi Zhu, Siliang Shao, Huafeng Pan, Zhongliang Cheng, Xin Rui

Mol Med Rep 17: 4803–4810, 2018; DOI: 10.3892/mmr.2018.8417

Following the publication of this paper, it was drawn to the Editor's attention by a concerned reader that certain of the cell migration and invasion assay data shown in Fig. 5C were strikingly similar to data appearing in different form in other articles by different authors at different research institutes, some of which have been retracted. Owing to the fact that the contentious data in the above article were already under consideration for publication, or had already been published, prior to its submission to *Molecular Medicine Reports*, the Editor has decided that this paper should be retracted from the Journal. After having been in contact with the authors, they agreed with the decision to retract the paper. The Editor apologizes to the readership for any inconvenience caused.

